# Nurses’ Perceptions of Individualized Care for Older Adults in the Post-COVID-19 Era: The Role of Ethical Workplace Climate

**DOI:** 10.3390/healthcare14152274

**Published:** 2026-07-25

**Authors:** Aurora García-Camacha Gutiérrez, Irene García-Camacha Gutiérrez, Riitta Suhonen, Beatriz Rodriguez-Martín

**Affiliations:** 1 Social and Health Research Center, University of Castilla-La Mancha, 16002 Cuenca, Spain; aurora.garciacamacha@alu.uclm.es; 2 Department of Mathematics, University of Castilla-La Mancha, 13071 Ciudad Real, Spain; 3 Department of Nursing Science, University of Turku, 20014 Turku, Finland; riisuh@utu.fi; 4 Turku University Hospital, The Wellbeing Services County of Southwest Finland, 20014 Turku, Finland; 5 Department of Nursing and Physiotherapy, Faculty of Health Sciences, University of Castilla-La Mancha, 45600 Talavera de la Reina, Spain; beatriz.rmartin@uclm.es

**Keywords:** nursing professional, individualized care, older adults, COVID-19

## Abstract

**Highlights:**

**What are the main findings?**

- Nurses reported an overall positive perception of individualized care for older adults in Spain.
- The highest scores were found in aspects related to patients’ clinical condition, while lower scores were observed in hygiene organization and the incorporation of patient preferences.
- A moderate, positive, and statistically significant association was found between workplace ethical climate and individualized nursing care.
- Nurses working in hospital settings perceived higher levels of individualized care than those working in nursing homes.

**What are the implications of the main findings?**

- A positive ethical workplace climate may enhance nurses’ ability to provide individualized care to older adults.
- Healthcare organizations should strengthen institutional support and promote ethical environments that facilitate individualized nursing practice.
- Greater interprofessional collaboration, particularly between nurses and physicians, is needed to support individualized clinical decision-making.
- Specific strategies and training programs should be implemented in long-term care settings to improve the integration of patient preferences and the consistency of individualized care.

**Abstract:**

Background/Objectives: Individualized nursing care is a fundamental component of high-quality healthcare, particularly in geriatric populations. However, its consistent implementation remains challenging, a situation further exacerbated by the COVID-19 pandemic due to increased workloads and resource constraints. This study aimed to assess nurses’ perceptions of individualized care for older adults in Spain and to examine the influence of the workplace ethical climate on these perceptions. Methods: A cross-sectional observational study was conducted using two validated instruments: the Individualized Care Scale–Nurse version (ICS-Nurse) and the Hospital Ethical Climate Survey (HECS). Descriptive and inferential analyses were performed to assess individualized care, ethical workplace climate, and the associations between both constructs. Results: A total of 96 nursing professionals participated in the study. Participants reported an overall favorable perception of individualized care. The highest scores were observed in the clinical situation dimension, whereas lower scores were reported for aspects related to hygiene organization and the incorporation of patients’ preferences. A moderate, positive, and statistically significant association was observed between ethical workplace climate and individualized care, particularly in dimensions related to patients and physicians. Participants working in hospital settings reported higher recent-experience individualized care scores than those working in nursing homes. Conclusions: These results highlight the critical role of an ethical workplace climate in supporting nurses’ capacity to deliver individualized care. They also underscore the need for targeted strategies to strengthen organizational support, enhance interprofessional collaboration—particularly with physicians—and promote training in individualized care practices. Addressing these factors may facilitate more consistent and comprehensive individualized care, especially in long-term care settings where structural challenges persist.

## 1. Introduction

It is well-known that nursing professionals must adapt their interventions to the specific needs of everyone [1]; individualized care is the best practice to ensure quality care [2]. Individualized care has been defined as care that considers the personal needs of patients, their clinical conditions, personal histories, and preferences, with the aim of promoting their maximum participation in clinical decision-making [3]. Its implementation in clinical practice and care plans is not yet complete, so further studies are needed to analyse care practices [1], the healthcare resources used [4], and the policy strategies of healthcare systems according to the health situation at any given time [5].

The COVID-19 pandemic substantially disrupted the organization and delivery of healthcare, with particularly severe consequences for older adults and long-term care facilities [6,7,8]. Nursing professionals experienced increased workloads, staff and resource shortages, emotional exhaustion, and ethical conflicts, all of which may have reduced their ability to adapt care to each older adult’s clinical condition, personal life situation, preferences, and participation in decision-making [9,10,11,12,13,14]. These pressures were especially relevant in older-adult care, where rapidly changing protocols and limited organizational resources created tensions between infection control, patient autonomy, and attention to individual needs [11,13,15,16,17].

Although the acute phase of the pandemic has ended, it remains important to determine whether its organizational and professional consequences continue to influence individualized care. In this context, examining nurses’ perceptions not only of individualized care but also of the ethical climate of their workplaces may help identify the relational and organizational conditions that support or hinder the provision of individualized care to older adults.

Providing individualized care increases nurses’ self-esteem, which improves the quality of care [18]. Furthermore, nurses with a lower workload and higher job satisfaction perceive that the care provided is more adapted to individual needs [19,20]. On the other hand, a lack of job motivation reduces personalised attention and, therefore, the quality of care [20]. Therefore, it is crucial to promote interventions that alleviate nurses’ workload, increase their job satisfaction, and ensure that patients’ real needs are met [21]. To this end, it is necessary to understand the personal, emotional, and work factors perceived by healthcare professionals [22] for the implementation and improvement of individualized care [23].

Currently, there is a need to use new tools to understand and develop the areas where patients and healthcare professionals perceive less personalised attention and to identify the elements that may influence this [24]. For this reason, in this study, the perception of nursing professionals of individualized care will be analysed using the Individualized Care Scale–Nurse version (ICS-nurse) [25]. In addition, to understand the possible work factors that may affect individualized care, the Hospital Ethical Climate Survey (HECS), which evaluates the work climate in hospitals and healthcare settings, will be used [26].

Despite the broad recognition of individualized care as a fundamental component of high-quality nursing care, its consistent implementation across healthcare settings remains challenging. The recent literature indicates that substantial gaps persist between the conceptual endorsement of care tailored to each individual and its routine application, particularly regarding the incorporation of older adults’ preferences, personal circumstances, everyday habits, and priorities into care planning and delivery [27]. Evidence obtained from nursing-home residents before and after the COVID-19 pandemic also suggests that, although some dimensions of individualized care may have improved, considerable opportunities remain to strengthen the consideration of patients’ personal life situations, decisional control, family participation, and individual preferences [28].

The organizational consequences of the pandemic may continue to affect the delivery of individualized care. Persistent workforce challenges in long-term care—including burnout, staff shortages, inadequate working conditions, insufficient training, and limited employee participation in care-related decisions—may restrict nursing professionals’ ability to respond to older adults’ individual needs and preferences [29]. Nevertheless, limited evidence is available regarding nurses’ current perceptions of individualized care after the acute phase of the pandemic or potential differences between hospital and nursing-home settings [28,29].

The ethical workplace climate may be particularly relevant to understanding these differences. Ethical workplace climate refers to the organizational and relational conditions under which healthcare professionals identify, discuss, and respond to ethical issues, including their relationships with colleagues, patients, supervisors, physicians, and the healthcare institution. Recent studies have associated a more positive ethical climate with better nursing task performance, critical thinking, professional values, and job satisfaction [30,31]. Furthermore, research on the care of older adults during the COVID-19 pandemic identified recurring tensions between infection-control measures and respect for patients’ autonomy, individual needs, communication, and equitable access to care, highlighting the importance of organizational support for ethically appropriate nursing practice [32].

However, previous studies have mainly examined ethical workplace climate in relation to professional outcomes, job satisfaction, or general nursing performance, rather than its association with individualized care for older adults. Consequently, it remains unknown whether a more supportive ethical workplace climate is associated with nurses’ greater perceived ability to consider older adults’ clinical conditions, personal life situations, preferences, and participation in care-related decision-making in the post-COVID-19 era. Evidence examining individualized care and ethical workplace climate simultaneously is particularly limited in Spain and across different care settings. Addressing this gap is important because organizational relationships, institutional policies, interprofessional collaboration, and opportunities for ethical deliberation may facilitate or constrain nurses’ ability to provide individualized care in everyday practice [30,31].

Therefore, this study contributes new evidence by jointly examining nurses’ perceptions of individualized care for older adults and the ethical climate of their workplaces in the post-COVID-19 context. It also examines the associations between the different dimensions of both constructs and explores potential differences between hospital and nursing-home settings. By integrating these perspectives, this study seeks to identify the organizational and relational factors that may support or hinder individualized nursing care and to inform targeted strategies for strengthening ethical workplace environments and the provision of individualized care to older adults.

Accordingly, this study aimed to assess nursing professionals’ perceptions of individualized care for older adults in Spain in the post-COVID-19 context and to examine the association between individualized care and the ethical climate of their workplaces. As secondary objectives, the study sought to identify the dimensions of individualized care and ethical workplace climate requiring greater improvement and to explore whether perceptions of individualized care differed according to participants’ sociodemographic and occupational characteristics, particularly between hospital and nursing-home settings.

## 2. Materials and Methods

### 2.1. Design

A cross-sectional observational study was conducted to analyse the perception of individualized care from the nurses’ perspective. Data were collected from June 2021 to June 2024. The extended recruitment period was required because the study targeted a specific professional population, participation was voluntary and non-incentivized, and the online survey was distributed through social media and professional networks rather than through a predefined sampling frame. To increase participation and broaden the geographical reach of the study, the survey was periodically recirculated through the same dissemination channels. The eligibility criteria, questionnaires, and data-collection procedures remained unchanged throughout the recruitment period. Each participant completed the questionnaire once, and no longitudinal follow-up was conducted.

First, a data collection methodology based on the use of digital technologies was implemented to gather the research sample and obtain participation from across the country. Specifically, an online survey was conducted and disseminated through various social media platforms and through professional nursing organizations and institutions, which received digital flyers to support dissemination. This strategy enabled broad national reach and enhanced sample diversity. The survey was hosted on Qualtrics, which was configured to reduce the likelihood of duplicate participation, while ensuring full anonymity and avoiding the collection of any directly identifying information.

### 2.2. Inclusion and/or Exclusion Criteria

Convenience sampling was used to select study participants, choosing individuals who voluntarily agreed to participate in the study and met the inclusion criteria: (1) being a nursing professional, (2) working in Spain in the field of elderly care, (3) volunteering to participate in the research, and (4) providing written informed consent. The exclusion criterion was not being actively employed at the time of data collection.

### 2.3. Instrument with Validity and Reliability

Before data collection, participants were informed about the nature of the study, the absence of adverse effects from their participation, and the confidentiality of the information.

Once participants provided their informed consent, sociodemographic variables were collected through a specially designed questionnaire, and they were then invited to complete the following questionnaires: Individualized Care Scale–Nurse (ICS-nurse) and Hospital Ethical Climate Survey (HECS).

The sociodemographic variables collected were age, sex, highest educational level (vocational training, diploma, degree, graduate, master’s, doctorate), work organisation in the centre (by tasks, by number of patients, and by cases), job position (nursing care assistant, nurse, supervisor, and director), specialised training in elderly care (specialisation in geriatric nursing, master’s in geriatrics, courses related to elderly care, or no specialised training in elderly care), years of experience in elderly care, number of patients under their care on the last working day, type of employment (permanent, interim, or temporary), type of institution where they work (public, private, or semi-public), workplace (nursing home, day centre, hospital, or other), and, specifically, the impact of COVID-19 on their professional work (positive, negative, or no impact).

The Individualized Care Scale–Nurse (ICS-nurse) is a validated scale in Spanish, designed to understand nursing professionals’ perceptions of providing individualized care [25]. It consists of two parts that examine two different times of providing individualized care: usual practice and recent experience. The scales comprise 17 items subdivided into 3 main blocks that primarily analyse: clinical situation, personal situation, and decision-making control. Both scales collect perceptions through the following Likert-type scale: (1) strongly disagree, (2) disagree, (3) neither agree nor disagree, (4) agree, and (5) strongly agree.

Additionally, the Hospital Ethical Climate Survey (HECS) will be used to analyse the association between the work environment and their evaluations of providing individualized nursing care. HECS includes 26 items scored on a Likert-type scale, assessing the quality of relationships with colleagues, patients, managers, the hospital, and doctors. The higher the score, the more positive the ethical environment of the workplace. This scale is validated and has been used in several countries [26].

### 2.4. Data Analysis

An exploratory and an inferential analysis were performed using the software Statistical Package for Social Sciences v.29 (SPSS 29). The demographic characteristics of the participants, the ICS-nurse and the HECS scores were first studied through a descriptive analysis. The internal consistency of the ICS-Nurse and HECS scores in the present sample was assessed using Cronbach’s alpha and McDonald’s omega. Reliability coefficients were calculated for the total ICS-A and ICS-B scores, their respective dimensions, and the total and dimensional HECS scores. Values of approximately 0.70 or higher were considered indicative of acceptable internal consistency. The Wilcoxon signed-rank test and the median test were the nonparametric test selected for exploring the discrepancies between numerical scores and factors with two or more categories, respectively. The ordinal nature of Likert scale data implies that the assumptions of parametric tests, such as normality of distribution or equality of variances, are not always applicable [33]. Thus, non-parametric tests provide robust decisions with ordinal data, such as ICS and HECS scores. For studying potential relationships between ICS and HECS scores, Spearman’s rank correlation coefficient and its corresponding hypothesis test were obtained. They are also indicated for ordinal-type data sets. The following values will be considered for the Spearman correlation coefficient (in absolute terms): ≤0.39 negligible or weak correlation, 0.40–0.69 moderate correlation, and ≥0.70 strong or very strong correlation [34]. To infer this, a contrast of the hypothesis is done so that only a couple of variables with strong or moderate association were studied (r_S ≥ 0.4 and *p* ≤ 0.05). All statistical evaluations were considered significant at the level of 0.05.

### 2.5. Ethical Concerns

The study was approved by the Research Ethics Committee for Medicines of the Integrated Care Management of Ciudad Real (23 July 2019, Act 07/2019).

All participants completed the informed consent form after receiving detailed information about the project. This study complied with the Declaration of Helsinki and the current legislation on data protection (Organic Law 3/2018, of 5 December, on the Protection of Personal Data and guarantee of digital rights) [35].

## 3. Results

### 3.1. Sample Description

Table 1 summarizes the main demographic features of the participants. A total of 96 nursing professionals whose patients were elderly people participated in the study, of which 86 were females and 10 were males. The average age of participants was 42.4 (±11.7) years old, with an average experience of 13.5 (±10.5) years. Most of the respondents were nurses (60.4%) and nurse assistants (31.3%). Regarding academic education, 58% of the participants had a diploma or degree in nursing, 27% had professional training, and 10.4% had completed a master’s degree. Nevertheless, only 20.9% of the respondents had a master’s or EIR specialized in geriatric nursing, since the majority undertook training courses (55.2%) or nonspecific training (24%). Most professionals reported having a permanent contract (55.2%), followed by occasional (27%) and temporary (17.7%) contracts. The vast majority of participants worked in public institutions (84.4%), and only 8.3% and 7.3% performed their professional activities in private and chartered institutions, respectively. In total, 82.3% reported working in nursing homes, while 17.7% worked in hospitals. Regarding work organization, 46.9% of the participants organized work by tasks, 39.6% by the number of patients, and only 13.5% by cases. The number of patients under their care exhibited substantial variability among professionals, with an average of 49.4 patients and a standard deviation of 41.8. Most participants reported that the COVID-19 pandemic had negatively affected their professional work (65.6%), whereas 18.8% reported no effect and 15.6% reported a positive effect.

### 3.2. Internal Consistency of the Instruments

Both instruments showed satisfactory internal consistency in the present sample. The total ICS-A showed a Cronbach’s alpha of 0.905 and a McDonald’s omega of 0.912, while the corresponding coefficients for the total ICS-B were 0.928 and 0.935, respectively. Across the ICS-Nurse dimensions, Cronbach’s alpha ranged from 0.685 to 0.917 and McDonald’s omega ranged from 0.690 to 0.925. The total HECS showed a Cronbach’s alpha and McDonald’s omega of 0.954. Across the HECS dimensions, Cronbach’s alpha ranged from 0.824 to 0.953 and McDonald’s omega ranged from 0.832 to 0.955.

### 3.3. Individualized Care Comparing the Ordinary Practice and the Recent Experience (ICS-A vs. ICS-B)

**Figure 1:**
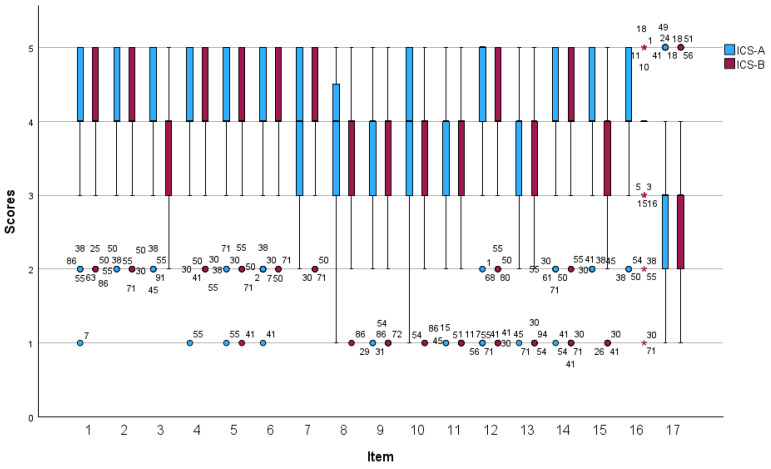
Description of the ICS-Nurse items. * represents extreme values, while the colored circles refer to outliers. The number appearing next to the outliers and extreme values is the subject identifier.

The median score was 4 out of 5 for most ICS-Nurse items (Table 2). The clinical situation dimension showed the highest mean scores (4.2 for ICS-A and 4.1 for ICS-B), followed by decisional control (3.9 and 3.7, respectively) and personal life situation (3.7 in both scales). Among the lower-scoring items were family involvement in care (3.5 for ICS-A and 3.6 for ICS-B) and the timing of washing according to patients’ preferences (2.7 and 2.6, respectively). The standard deviations of the item scores ranged from 0.7 to 1.2, so the results were consistent. The highest-scoring item concerned the provision of information in language that was easy for patients to understand (4.4 for ICS-A and 4.2 for ICS-B), whereas the lowest-scoring item concerned the timing of washing according to patients’ preferences (2.7 and 2.6, respectively). No statistically significant differences between the scores obtained in the ordinary practice (ICS-A) and the recent experience (ICS-B) were observed for most items. Statistically significant differences between ICS-A and ICS-B were observed for responsibility for care (item 3; *p* < 0.001), meaning of the illness (item 7; *p* = 0.028), personal wishes (item 14; *p* = 0.006), and decision-making (item 15; *p* < 0.001) (see Figure 1). Scores were lower for recent experience than for usual practice for responsibility for care, personal wishes, and decision-making, whereas the score for meaning of the illness was higher for recent experience. At the dimension level, decisional control was also lower for ICS-B than for ICS-A (3.7 vs. 3.9; *p* = 0.012). No statistically significant difference was found between the total ICS-A and ICS-B scores (*p* = 0.498).

### 3.4. Hospital Ethical Climate Survey (HECS)

**Figure 2:**
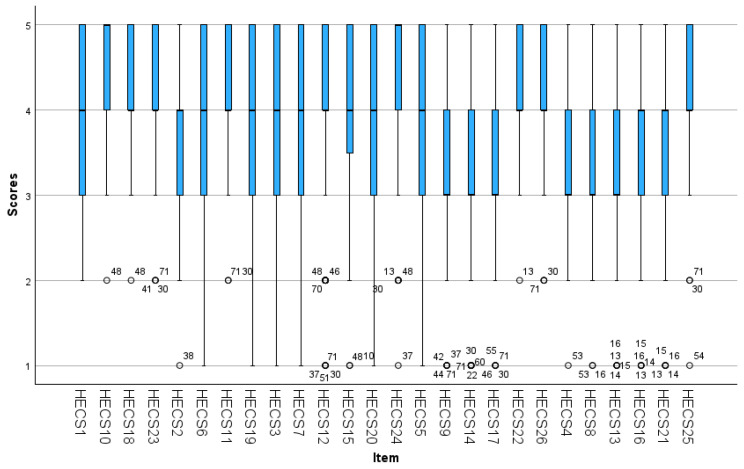
Description of the HECS items. The colored circles refer to outliers. The number appearing next to the outliers values is the subject identifier.

The median HECS score was 4 or higher for all dimensions except the care center dimension, which had a median score of 3.5 (Table 3). The workmates dimension showed the highest mean score (4.2), followed by the supervisor (4.0), patients (3.9), physicians (3.7), and care center dimensions (3.6). The highest-scoring items were item 10, concerning assistance from colleagues in difficult patient-care situations (mean = 4.4), and item 24, concerning respect for the supervisor (mean = 4.4)—see Figure 2. Three of the four lowest-scoring items belonged to the care center dimension. All of them were rated with an average score of 3.4 points and reflected perceptions about hospital policies (item 4), the role of professionals in the institution (item 8), and the consideration of feelings and values in conflict resolution (item 13). The remaining item with a mean score of 3.4 was item 14 in the physicians dimension, concerning nurses’ participation in treatment decisions. The item-level standard deviations ranged from 0.8 to 1.3.

### 3.5. Individualized Care vs. Demographics

To analyse potential variations in the scores obtained for individualized care and the different demographic factors collected, a study was conducted to determine whether the median scores of ICS-A and ICS-B differed significantly between groups (*p*-values can be found in Table 1). In the case of being a numerical factor, Spearman correlations (between brackets) and its corresponding hypothesis test were included in Table 1. No statistically significant associations were observed between the sociodemographic or occupational variables and ICS-A scores. For ICS-B, a statistically significant difference was observed according to workplace setting (*p* = 0.006). The median ICS-B score was 5 among participants working in hospitals and 4 among those working in nursing homes (*p* = 0.006).

### 3.6. Individualized Care vs. Hospital Ethical Climate

Table 4 presents the correlations between the HECS dimensions and the total ICS-A and ICS-B scores. The total HECS score showed a moderate positive correlation with both the total ICS-A score (r_s_ = 0.4, *p* < 0.001) and the total ICS-B score (r_s_ = 0.4, *p* < 0.001). Among the HECS dimensions, the strongest correlations were observed for patients and physicians. The patients dimension was positively correlated with the total ICS-A (r_s_ = 0.4, *p* < 0.001) and ICS-B scores (r_s_ = 0.5, *p* < 0.001). Similarly, the physicians dimension was positively correlated with the total ICS-A (r_s_ = 0.5, *p* < 0.001) and ICS-B scores (r_s_ = 0.4, *p* < 0.001). The correlations involving workmates, supervisors, and the care center did not reach the prespecified threshold of r_s_ ≥ 0.4.

The complete correlation matrix between all ICS-Nurse and HECS dimensions is provided in Supplementary Table S1. At the dimensional level, correlations meeting the prespecified criterion were observed primarily between the HECS patients and physicians dimensions and the ICS clinical situation and decisional control dimensions.

## 4. Discussion

This study examined nursing professionals’ perceptions of individualized care for older adults and their association with ethical workplace climate. Three principal findings emerged. First, participants reported relatively high scores for individualized care overall, although scores were higher for dimensions related to patients’ clinical situations than for personal life situations and decisional control. Second, ethical workplace climate was moderately and positively associated with individualized care, with the clearest associations involving the HECS patients and physicians dimensions. Third, participants working in hospitals reported higher recent-experience individualized care scores than those working in nursing homes. These findings should be interpreted as patterns of association rather than evidence of causal relationships due to the nature of the present studio.

The higher scores observed for the clinical situation dimension suggest that nursing professionals may find it easier to individualize care around directly observable clinical needs than around older adults’ everyday routines, personal circumstances, and participation in decision-making. Clinical assessment is embedded in standardized nursing processes and is generally supported by established protocols, documentation systems, and professional competencies. By contrast, incorporating personal preferences and everyday habits may require greater time, continuity, flexibility, knowledge of the individual, and coordination with families and other professionals. Recent research continues to show that individualized care is not implemented uniformly across its different dimensions and that personal life situations and decisional control remain more difficult to integrate into routine practice, particularly in care for older adults [28,36].

The comparatively lower scores for the consideration of patients’ preferences and for organizing washing according to individual wishes are consistent with broader international evidence showing that individualized care is often more difficult to implement in aspects that require negotiation, flexibility, and active patient participation. A recent qualitative meta-synthesis found that older adults’ expression of preferences and involvement in shared decision-making may be limited by communication difficulties, paternalistic professional attitudes, insufficient information, and organizational routines that leave little room for active participation [37]. In the present study, the particularly low score for preferred washing times may reflect the fact that hygiene care is commonly organized around fixed schedules, task allocation, staffing availability, and collective routines rather than residents’ preferred timing. This interpretation is supported by an international systematic review of residential care, which identified limited time, workload, staffing levels, insufficient resources, staff knowledge and skills, residents’ responses to care, and communication as relevant barriers to individualized hygiene practices [38]. These findings suggest that lower scores may not necessarily indicate that professionals attach little importance to individual preferences; rather, they may reflect practical tensions between respecting personal wishes and completing essential care within established institutional routines. From a practical perspective, facilities could explore more flexible hygiene schedules, document residents’ preferences systematically, and involve residents and families in care planning. However, intervention studies would be required to determine whether these strategies produce measurable improvements in individualized care.

The comparison between usual practice and recent experience provides a more nuanced pattern than an overall improvement or deterioration. No statistically significant difference was observed between the total ICS-A and ICS-B scores. However, recent-experience scores were lower for responsibility for care, personal wishes, and decision-making, while the score for meaning of illness was higher. These item-level differences may indicate that some components of individualized care are more sensitive than others to immediate workload, staffing, care routines, or organizational circumstances.

A central finding was the moderate positive association between ethical workplace climate and individualized care. Several mechanisms may plausibly contribute to this relationship. A supportive ethical climate may give nursing professionals greater confidence to express concerns, question routines that do not reflect patients’ needs, and advocate for older adults’ preferences. It may also strengthen professional autonomy by creating organizational conditions in which nurses’ knowledge and ethical judgments are acknowledged in care planning. Conversely, when organizational expectations are unclear, professionals’ views are not taken into account, or ethical concerns cannot be openly discussed, nurses may have less opportunity to adapt care to each patient, even when they recognize the need to do so. Recent evidence associates a more positive ethical climate with professional values, job satisfaction, critical thinking, and nursing task performance, although these studies are also predominantly observational and do not establish causal direction [30,31].

Interdisciplinary collaboration may represent another mechanism linking ethical workplace climate and individualized care. The associations involving the HECS physicians dimension suggest that individualized care may depend partly on whether nurses are heard, respected, and included in treatment decisions. Nurses often possess detailed knowledge of patients’ responses, preferences, daily routines, and psychosocial circumstances. When this knowledge is incorporated into interdisciplinary decision-making, care plans may be better adapted to the individual. By contrast, hierarchical communication or limited nurse participation may hinder the translation of such knowledge into clinical decisions. This interpretation does not imply that nurse–physician collaboration caused higher individualized care scores; rather, it identifies a plausible relational pathway that should be tested in longitudinal or intervention studies.

Leadership may also shape this association by determining whether ethical concerns are discussed openly, whether staff receive support when facing difficult decisions, and whether sufficient flexibility exists to adapt routines to individual needs. Supportive and relational leadership has been associated with trust, organizational commitment, empowerment, motivation, and nurses’ work-related well-being. These factors may increase professionals’ capacity to participate actively in decisions and advocate for individualized care. In contrast, leadership focused mainly on task completion may unintentionally reinforce standardized routines and reduce opportunities to incorporate patient preferences. A systematic review found that the relationships between nursing leadership and professional well-being may operate through trust, empowerment, organizational relationships, job satisfaction, and motivation [39].

Organizational culture provides a broader context for these mechanisms. An organizational culture that values learning, psychological safety, ethical reflection, and shared decision-making may make it easier for professionals to raise concerns and adapt established practices. Such a culture may be particularly relevant when individualized preferences conflict with institutional routines, resource constraints, or risk-management procedures. Ethical workplace climate should therefore not be understood merely as an individual attitude or a measure of job satisfaction, but as a relational and organizational context within which individualized care is negotiated and delivered. The relatively lower scores found for the HECS care center dimension—including institutional policies, shared organizational mission, and the consideration of different perspectives when resolving conflicts—suggest that organization-level conditions may warrant attention even when relationships with colleagues and supervisors are rated relatively positively.

The associations involving the HECS patients dimension may partly reflect conceptual proximity between the two instruments. HECS items concerning access to information, expectations, and respect for patients’ wishes overlap conceptually with aspects of the ICS-Nurse concerning information, preferences, and participation in decision-making. Some degree of correlation is therefore expected. However, the association may also indicate that environments in which patients’ wishes are recognized provide professionals with greater opportunities to individualize care. Future research should examine whether these associations remain after accounting for potentially overlapping content and should consider more complex models incorporating professional autonomy, ethical leadership, interdisciplinary collaboration, staffing, and workload as possible mediators or moderators.

Participants working in hospitals reported a higher median ICS-B score than those working in nursing homes. This difference deserves cautious interpretation. Hospitals and nursing homes differ in staffing structures, availability of clinical resources, professional mix, organizational routines, and access to medical support. Hospital teams may have more immediate access to multidisciplinary decision-making and diagnostic and therapeutic resources. Nursing homes, however, provide long-term and relational care that could theoretically facilitate knowledge of residents’ histories and preferences. At the same time, the potential benefits of continuity may be constrained by staffing shortages, task-oriented organization, high resident-to-staff ratios, and limited time to accommodate individual routines. The recent literature has emphasized continuing workforce pressures in long-term care, including staff shortages, burnout, inadequate working conditions, insufficient training, and limited employee participation in care planning [29]. Nevertheless, the hospital–nursing home comparison should be regarded as exploratory because the groups were markedly unequal: only 17 participants worked in hospitals, compared with 79 in nursing homes. The small hospital subgroup reduces the precision and stability of the estimate and increases the possibility that the observed difference reflects unmeasured differences in participant or workplace characteristics. Furthermore, workplace setting may be confounded by staffing, professional role, institutional ownership, workload, training, or patient characteristics. Then, present findings identify a setting-related difference that should be examined in larger studies using balanced or stratified samples and multivariable analyses.

The observed difference between hospital and nursing-home settings may also be influenced by participant- and organization-level factors. In the bivariate analyses, years of professional experience, educational level, and the number of patients under each professional’s care were not significantly associated with the ICS-Nurse scores. However, the absence of statistically significant bivariate associations does not exclude their potential role as confounding or interacting factors. For example, professional experience and education may influence nurses’ confidence, ethical reasoning, and participation in clinical decision-making, whereas workload may affect the time and flexibility available to consider patients’ preferences and everyday routines.

Organizational differences between facilities may also be relevant, including staffing arrangements, work organization, availability of multidisciplinary support, leadership practices, institutional policies, and access to resources. These characteristics were not examined in sufficient detail to determine whether they contributed to the observed setting-related difference or to the association between ethical workplace climate and individualized care. Future studies should collect facility-level information and use appropriately powered multivariable or multilevel analyses to distinguish the contribution of individual professional characteristics from that of the organizational context.

The COVID-19 context may have contributed to the organizational conditions considered in this study, but its role cannot be isolated from the present data. Recent systematic evidence documents substantial levels of burnout, anxiety, depression, acute stress, and post-traumatic stress symptoms among healthcare professionals during the pandemic, with nurses representing a large proportion of the populations studied [40]. These psychological pressures could plausibly influence professionals’ capacity to engage in complex communication, shared decision-making, and adaptation of care.

### Limitations

This study has several limitations. The use of convenience sampling and voluntary participation may have introduced self-selection bias, as professionals with a particular interest in individualized care or ethical workplace climate may have been more likely to participate. Moreover, the relatively small sample size (*n* = 96) and the predominance of nursing-home professionals may limit the representativeness and generalizability of the findings to the broader nursing population in Spain. Comparisons between hospital and nursing-home settings should also be interpreted cautiously because of the unequal group sizes.

Both individualized care and ethical workplace climate were assessed using self-reported measures, which may be affected by social desirability, recall bias, and common-method variance. The prolonged recruitment period, from June 2021 to June 2024, should also be considered, as participants completed the questionnaire at different time points and a possible influence of temporal or contextual factors can neither be confirmed nor excluded. Finally, the cross-sectional design only allows identification of associations between the variables and does not establish their temporal sequence, directionality, or causality. Therefore, the observed association between ethical workplace climate and individualized care should not be interpreted as evidence of a cause-and-effect relationship. Future studies should use larger and more representative samples and longitudinal or intervention designs to further examine these findings.

Although years of professional experience, educational level, and number of patients were examined in the bivariate analyses, the relatively small sample precluded a comprehensive multivariable assessment of potential confounding. In addition, relevant facility-level characteristics—such as staffing patterns, work organization, leadership practices, institutional policies, and resource availability—were not assessed in sufficient detail. Residual confounding by individual and organizational factors should be considered in future studies.

## 5. Conclusions

Nursing professionals reported favorable perceptions of individualized care for older adults, with higher scores for aspects related to patients’ clinical situations and lower scores for personal life situations and decisional control. A positive association was observed between ethical workplace climate and individualized care, particularly in dimensions concerning relationships with patients and physicians. These findings identify professional relationships, interdisciplinary collaboration, and the organizational environment as potentially relevant factors in the provision of individualized care. Healthcare organizations may therefore consider exploring strategies aimed at strengthening ethical workplace environments, professional participation, and collaboration between nurses and physicians.

## Figures and Tables

**Table 1:** Description of the sociodemographic variables of the sample.

*n* = 96	Statistics	$p_{A}$	$p_{B}$
Age Mean (SD)	42.4 (±11.7)	0.614 (0.1)	0.402 (0.1)
Gender		0.268	0.935
Male	10 (10.4%)		
Female	86 (89.6%)		
Working experience (in ages)	13.5 (±10.5)	0.131 (0.2)	0.494 (0.1)
Number of patients	49.4 (±41.8)	0.271 (0.1)	0.726 (0)
Job position		0.557	0.717
Nurse Assistant	30 (31.3%)		
Nurse	58 (60.4%)		
Nurse Supervisor	7 (7.3%)		
Director	1 (1.0%)		
Academic Education		0.211	0.882
Assistant Nursing professional training	26 (27.1%)		
Diploma in Nursing	37 (38.5%)		
Degree in Nursing	19 (19.8%)		
Bachelor’s degree in Nursing	2 (2.1%)		
Master’s in Nursing	10 (10.4%)		
Others	2 (2.1%)		
Speciality in Geriatrics		0.537	0.325
None	23 (24.0%)		
Training courses	53 (55.2%)		
Master	6 (6.3%)		
EIR—geriatric nursing	14 (14.6%)		
Type of contract		0.655	0.776
Permanent contract	53 (55.2%)		
Temporary contract	17 (17.7%)		
Occasional contract	26 (27.1%)		
Type of work organization		0.140	0.107
By cases	13 (13.5%)		
By tasks	45 (46.9%)		
By patient number	38 (39.6%)		
Type of institution		0.227	0.375
Public	81 (84.4%)		
Private	8 (8.3%)		
Chartered	7 (7.3%)		
Institution		0.427	0.006
Hospital	17 (17.7%)		
Nursing home	79 (82.3%)		
COVID-19 pandemic affectation		0.128	0.710
Positively	15 (15.6%)		
Negatively	63 (65.6%)		
No affectation	18 (18.8%)		

Statistics: For categorical variables, the number of subjects and its corresponding percentage (in brackets) is reported, while the average and its standard deviation (in brackets) is reported for numerical variables. Abbreviations: $p_{A}$ and $p_{B}$: *p*-value resulting from the Wilcoxon signed-rank test and median test for ICS-A and ICS-B scores and sociodemographic factors with two or more categories, respectively. In the case of numerical sociodemographic variables, Spearman’s correlation coefficient (in brackets) and the *p*-value corresponding to its hypothesis test are given.

**Table 2:** Description of ICS-Nurse items.

	ICS-A-Nurse			ICS-B-Nurse			
Item Content	Mean (±SD)	Median	Range	Mean (±SD)	Median	Range	p
Clinical situation	4.2 (±0.4)	4	1–5	4.1 (±0.4)	4	1–5	0.514
1. Feelings	4.2 (±0.9)	4	1–5	4.1 (±0.8)	4	2–5	0.689
2. Needs for care	4.3 (±0.8)	4	2–5	4.3 (±0.8)	4	2–5	0.853
3. Responsibility for care	4.2 (±0.8)	4	2–5	3.7 (±0.9)	4	2–5	<0.001
4. Changes in conditions	4.3 (±0.7)	4	1–5	4.1 (±0.8)	4	2–5	0.101
5. Fears and anxieties	4.1 (±0.8)	4	1–5	4.2 (±0.8)	4	1–5	0.826
6. Effects of the illness	4.1 (±0.9)	4	1–5	4.2 (±0.7)	4	2–5	0.197
7. Meaning of the illness	3.9 (±0.9)	4	2–5	4.1 (±0.8)	4	2–5	0.028
Personal life situation	3.7 (±0.6)	4	1–5	3.7 (±0.6)	4	1–5	0.724
8. Activities in daily life	3.9 (±0.9)	4	1–5	3.8 (±0.8)	4	1–5	0.199
9. Hospital experience	3.6 (±1.1)	4	1–5	3.6 (±1.0)	4	1–5	0.641
10. Everyday habits	3.8 (±1.0)	4	1–5	3.8 (±0.9)	4	1–5	0.665
11. Family in care	3.5 (±0.9)	4	1–5	3.6 (±1.0)	4	1–5	0.272
Decisional control	3.9 (±0.9)	4	1–5	3.7 (±0.8)	4	1–5	0.012
12. Information	4.4 (±0.9)	5	1–5	4.2 (±0.9)	4	1–5	0.056
13. Want to know	3.8 (±0.9)	4	1–5	3.8 (±1.0)	4	1–5	0.797
14. Personal wishes	4.2 (±0.9)	4	1–5	4.0 (±0.9)	4	1–5	0.006
15. Decision making	4.2 (±0.9)	4	2–5	3.8 (±1.0)	4	1–5	<0.001
16. Express opinion	4.0 (±0.8)	4	2–5	4.0 (±0.8)	4	1–5	0.315
17. Want to wash	2.7 (±1.2)	3	1–5	2.6 (±1.1)	3	1–5	0.314
Total	4.0 (±0.8)	4	1–5	3.9 (±0.7)	4	1–5	0.498

Abbreviations: ICS-A: Individualized Care Scale-Nurse Scale A; ICS-B: Individualized Care Scale-Nurse Scale B; SD: Standard Deviation; p: *p*-value resulting from the Wilcoxon signed-rank test comparing ICS-A and ICS-B scores. © This instrument has copyright reserved. Riitta Suhonen 2007.

**Table 3:** Description of Hospital Ethical Climate Survey (HECS).

	HECS Scores		
Item Content	Mean (±SD)	Median	Range
Workmates	4.2 (±0.5)	4.5	2–5
1. Peers listen	4.0 (±0.8)	4	2–5
10. Peers help	4.4 (±0.8)	5	2–5
18. Competent colleagues	4.2 (±0.8)	4	2–5
23. Safe patient care	4.3 (±0.8)	4	2–5
Patients	3.9 (±0.6)	4	1–5
2. Patients know what to expect	3.6 (±0.9)	4	1–5
6. Access to information	4.0 (±1.0)	4	1–5
11. Use information	4.1 (±0.8)	4	2–5
19. Patients’ wishes respected	3.8 (±1.1)	4	1–5
Supervisor	4.0 (±0.4)	4	1–5
3. Manager helps me	3.7 (±1.3)	4	1–5
7. Manager supports me	3.8 (±1.2)	4	1–4
12. Manager listens to me	4.0 (±1.2)	4	1–4
15. Manager someone to trust	4.0 (±1.1)	4	1–5
20. Manager helps peer decide	4.0 (±1.1)	4	1–5
24. Manager someone I respect	4.4 (±0.9)	5	1–5
Physicians	3.7 (±0.6)	4	1–5
5. Nurse, physicians trust one another	3.8 (±0.9)	4	1–5
9. Physicians ask nurses’ opinions	3.5 (±1.0)	3	1–5
14. I participate in treatment decisions	3.4 (±1.2)	3	1–4
17. Nurses, physicians respect other’s opinions	3.6 (±1.1)	3	1–5
22. Nurses, physicians respect each other	4.2 (±0.8)	4	2–5
26. Nurses supported in hospital	4.1 (±0.8)	3	2–5
Care Center	3.6 (±0.5)	3.5	1–5
4. Hospital policies help me	3.4 (±1.0)	3	1–5
8. Hospital’s mission shared	3.4 (±1.0)	3	1–5
13. Everyone’s feelings taken into account	3.4 (±0.9)	3	1–5
16. Conflict dealt with openly	3.5 (±1.0)	3.5	1–5
21. Sense of questioning, learning	3.6 (±1.0)	4	1–5
25. Practice nursing way I believe it should be	4.1 (±1.0)	4	1–5
Total	3.9 (±0.8)	4	1–5

Abbreviations: SD: Standard Deviation.

**Table 4:** Description of Individualized care vs. hospital ethical climate.

ICS Dimension	Total			
HECS	$r_{A}$	$p_{A}$	$r_{B}$	$p_{B}$
Workmates	0.3	0.016	0.3	0.004
Patients	**0.4**	**<0.001**	**0.5**	**<0.001**
Supervisor	0.2	0.033	0.3	0.002
Physicians	**0.5**	**<0.001**	**0.4**	**<0.001**
Care Centre	0.1	0.159	0.2	0.023
Total	**0.4**	**<0.001**	**0.4**	**<0.001**

Abbreviations: r_A and r_B: Spearman’s correlation coefficient of the ICS-A and ICS-B scores and the corresponding HECS dimension; p_A and p_B: *p*-values corresponding to Spearman’s hypothesis test using ICS-A and ICS-B scores, respectively. Spearman’s rank correlation coefficients are reported. Coefficients meeting the prespecified criterion for a statistically significant moderate or stronger association (r_s_ ≥ 0.4 and *p* ≤ 0.05) are shown in bold. ICS-A reflects nurses’ perceptions of support for individualized care in usual practice, whereas ICS-B reflects perceptions of individualized care provided in recent practice.

## Data Availability

The data supporting the findings of this study are available from the corresponding author upon reasonable request. The data are not publicly available due to participant privacy and ethical restrictions.

## Supplementary Materials

The following supporting information can be downloaded at: https://www.mdpi.com/article/10.3390/healthcare14152274/s1, Table S1: Correlations between the dimensions of the Individualized Care Scale–Nurse and the Hospital Ethical Climate Survey.

## Abbreviations

The following abbreviations are used in this manuscript:

ICS-nurse	Individualized Care Scale–Nurse
HECS	Hospital Ethical Climate Survey
