# Supplementary material for: Nurses’ Perceptions of Individualized Care for Older Adults in the Post-COVID-19 Era: The Role of Ethical Workplace Climate
Source: Healthcare (Basel). 2026 Jul 25;14(15):2274. doi: 10.3390/healthcare14152274 (PMC13463863; doi:10.3390/healthcare14152274)
Supplement: Supplementary file 1 [file healthcare-14-02274-s001.zip › healthcare-4423974-supplementary.pdf]

Table S1. Correlations between the dimensions of the Individualized Care Scale–Nurse and the Hospital Ethical Climate Survey.

ICS Dimension	**Clinical situation**				**Personal fife situation**				**Decisional control**				**Total**			
HECS	r_A	p_A	r_B	p_B	r_A	p_A	r_B	p_B	r_A	p_A	r_B	p_B	r_A	p_A	r_B	p_B
**Workmates**	0.3	0.003	**0.4**	**<0.001**	0.3	0.006	0	0.8	0.1	0.170	0.2	0.077	0.3	0.016	0.3	0.004
**Patients**	**0.4**	**<0.001**	**0.5**	**<0.001**	0.3	0.005	0.2	0.118	**0.4**	**<0.001**	**0.4**	**<0.01**	**0.4**	**<0.001**	**0.5**	**<0.001**
**Supervisor**	0.2	0.089	0.3	0.002	0.3	0.005	0.1	0.224	0.3	<0.001	0.3	0.002	0.2	0.033	0.3	0.002
**Physicians**	**0.5**	**<0.001**	**0.4**	**<0.001**	**0.5**	**<0.001**	0.3	0.005	**0.4**	**<0.001**	0.3	0.003	**0.5**	**<0.001**	**0.4**	**<0.001**
**Care Centre**	0.1	0.199	0.2	0.04	0.2	0.021	0.1	0.431	0.3	0.004	0.3	0.010	0.1	0.159	0.2	0.023
**Total**	0.3	<0.001	**0.4**	**<0.001**	**0.4**	**<0.001**	0.2	0.070	**0.4**	**<0.001**	**0.4**	**<0.001**	**0.4**	**<0.001**	**0.4**	**<0.001**

Abbreviations: r_A and r_B: Spearman's correlation coefficient of the ICS-A and ICS-B scores and the corresponding HECS dimension; p_A and p_B: p-values corresponding to Spearman's hypothesis test using ICS-A and ICS-B scores, respectively. Values **in bold** highlight the verification of the criterion explained in *Statistical Analysis* section.
